# A home fitness satisfaction model for Chinese residents during the COVID-19 pandemic based on SEM analysis

**DOI:** 10.3389/fpubh.2022.947223

**Published:** 2022-09-27

**Authors:** Dexu Chen, Bin Zhang

**Affiliations:** ^1^School of Physical Education, Shandong University, Jinan, China; ^2^Zhejiang College of Construction, Hangzhou, China

**Keywords:** home fitness, satisfaction, healthcare, structural equation, COVID-19

## Abstract

In response to the COVID-19 epidemic, China proposes to take this epidemic as an opportunity to further cultivate residents' healthy living habits and meet their demand for healthy living. Based on relevant research reviews and theoretical analysis, the concept model of home fitness satisfaction for Chinese residents is constructed. The research hypothesis is tested by SEM, and the conceptual model is modified. Finally, the model of home fitness satisfaction for Chinese residents is obtained. The results show that during the epidemic period, Chinese residents' enthusiasm for home fitness remains unchanged, online services provide powerful social support, and the home fitness environment still has an enhanced space.

## Problem statement

The COVID-19 epidemic broke out all over the world, which imposed a near-total lockdown on citizens in China. In this war without smoke, staying at home played an important part. During the epidemic period, the General Office of the State General Administration of Sport issued the notice on vigorously promoting scientific home-based fitness methods, requiring local sports departments to actively promote scientific home-based fitness and promote people's health with the cooperation of local party committees and governments. Media platforms at all levels and of all kinds have launched home-based fitness programs, and home-based fitness has become a hot topic. The number of search results for “epidemic home-based fitness” and “epidemic home-based exercise” on Baidu was 18,300,000 and 43,900,000, respectively. On the one hand, the outbreak of the epidemic caused great mental trauma for people, forcing people to re-examine the importance of a healthy lifestyle, which helps people make greater progress between the residents of sports and healthy cognition. Meanwhile, the value function of sports fitness to improve people's physical fitness and promote physical and mental health is manifested again in this special period. On the other hand, affected by the epidemic, sports departments at all levels, schools, as well as sports and fitness institutions have launched online fitness courses and sports and fitness live broadcasts. Online fitness clocking and other services have attracted high attention. However, the flash-in-the-pan craze of home fitness is not what society needs. How to continue to promote home fitness services, how to make more people more convenient to participate in in-home fitness, and how to make use of Internet technology to promote the reform of social sports services are the key points of our further study. Therefore, it is necessary to study home fitness from the perspective of residents' satisfaction to enhance the effect of home fitness, strengthen the weak links of home fitness, and promote the health of the people. Based on the investigation of residents' satisfaction with home fitness and its influencing factors during the epidemic period, this study builds a model of residents' satisfaction and analyzes the influencing factors of residents' home fitness during the epidemic period to improve the effect of home fitness and promote national fitness.

Infection with novel coronavirus pneumonia does not necessarily have a physical impact, as in a latent phase, but if it is in the onset phase, the condition is of varying severity, and the physical impact is different and cannot be generalized. (1) Latency. If the following infection with novel coronavirus pneumonia is found at a latent stage of the condition, usually without obvious symptoms, it may be detected at the time nucleic acid testing is performed, at which point there will be no apparent effect on the body. (2) Onset stage. Less severe: if the new coronavirus pneumonia develops after the onset, the condition is relatively mild and may present with fever, dry cough, and other symptoms, but also with generalized weakness, some people will have symptoms such as runny nose and sneezing, should actively visit the hospital for supportive care, make appropriate exercise to improve their physical fitness, the condition generally recovers slowly and does not cause many effects on their physical health; Severe disease: if the new coronavirus pneumonia continues to progress, is not treated promptly, causes more damage to the body, may present with symptoms such as dysgeusia, anosmia, or even dyspnea, and some may present with hypoxemia, sepsis, shock, or acute respiratory distress syndrome, as well as multiple organ failure.

## Model construction and research hypothesis

The study of “satisfaction” is coined in the study of “customer satisfaction” in economic problems. It is generally believed in the academic area that customer satisfaction is the psychological reaction of customers after their needs are met, and it is the evaluation of customers of the product or the degree to which it meets their own needs (1). In the 1990's, the American Customer Satisfaction Model (ACSI) and the European Customer Satisfaction Model (ECSI) were widely adopted, and in the 21st century, the Chinese Customer Satisfaction Model (CCSI) was created (2). The satisfaction model can reveal the relationship between various factors affecting satisfaction, and a structural equation is often used to verify and adjust the satisfaction model (3). Structural equations provide a rigorous scientific procedure for quantitative research of abstract concepts and enable researchers to test research hypotheses through scientific statistical analysis. In this study, structural equations were used to verify and build a satisfaction model, and the residents' satisfaction with home fitness and its influencing factors were included in the same model, and the relationship among multiple factors was processed and analyzed at the same time. Compared with the simple descriptive analysis, the structural equation model can better explain the residents' satisfaction with home fitness and its influencing factors during the epidemic period.

### Conceptual model construction

**Figure 1:**
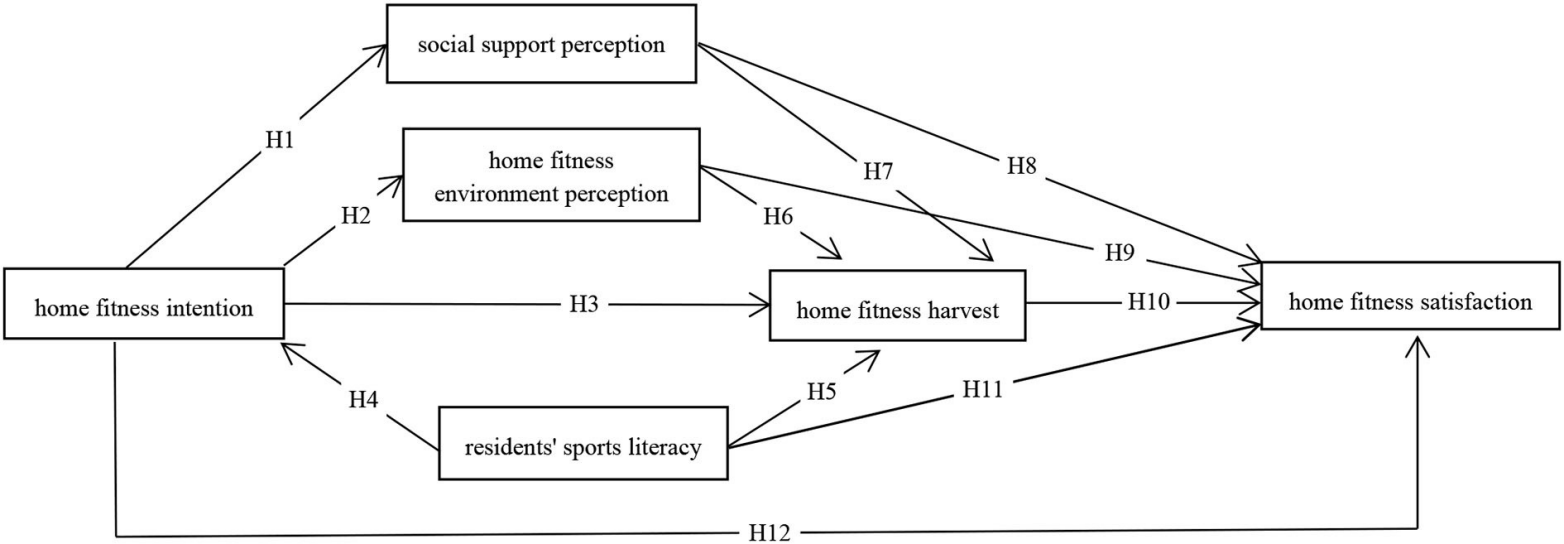
Conceptual model of residents' satisfaction with home fitness during the epidemic period.

Through comparative analysis, the American customer satisfaction model and China customer satisfaction index (ACSI) model (CSCI) in the “customer perceived value (4), customer expectations, and quality awareness,” are closer to the residents' home fitness satisfaction variables such as model building, so this study is based on ACSI and CSCI built during the outbreak of residents' fitness satisfaction model at home. In the model, the intention to exercise at home, the perception of social support, the perception of the environment of exercise at home, the residents' physical literacy, and the gain of exercise at home are the cause variables, while the residents' satisfaction with exercise at home is the target variable. Figure 1 is the conceptual model of residents' satisfaction with home fitness during the epidemic period.

The concept of home fitness intention comes from the survey of residents' sports awareness and draws on the concept of “customer expectation,” which is the expectation of customers for the products or services that will happen. The intention of exercising at home during the epidemic period refers to the residents' expectation to participate in the exercise at home during the epidemic period, including the idea of exercising at home, the desire to exercise with others, the expectation to bring pleasure to the exercise at home, and the expectation to release pressure through the exercise at home (5). Previous studies have shown that residents' intention to exercise at home has a positive impact on satisfaction with exercise at home, perception of social support for sports, and perception of support for sports environment. This study assumes that residents' intention to exercise at home has a positive impact on their perception of social support and fitness environment at home, residents' physical literacy, and residents' satisfaction with sports (6).

The perception of social support refers to the quality perception in the customer satisfaction model, which refers to the subjective feelings of customers when they receive products or services. In this study, the perception of social support refers to the support that residents received from the government, schools, media, and other platforms on home fitness during the period of the epidemic, as well as the online or offline home fitness guidance from others. This support can be either physical, such as mailing fitness books and equipment, or virtual, such as online guidance and a fitness clock. In this study, the perception of social support was mainly assessed by residents' evaluation of the above support methods and contents. In previous studies, researchers believe that quality perception directly affects customers' evaluation of products or services and is an important factor affecting customer satisfaction. Therefore, this study hypothesizes that the perception of social support has a positive impact on residents' sports harvest and satisfaction with sports (7).

The perception of a home fitness environment also comes from the concept of “quality perception.” Home fitness environment perception refers to residents' evaluation of the size and comfort of the home fitness space, the type and quantity of equipment, and the home fitness atmosphere during their stay at home. The home-based fitness environment is the material guarantee for residents to participate in home-based fitness, which affects residents' home-based fitness to a great extent (8). This study hypothesizes that the perception of the home fitness environment plays a positive role in residents' home fitness harvest and satisfaction with home fitness.

Residents' physical literacy mainly refers to the acquired physical skills, physical knowledge, and physical behavior (9). To a certain extent, residents' physical literacy reflects their ability to participate in physical activities as well as their frequency of participation in physical activities. It is also an important indicator of whether residents can carry out independent scientific fitness tests. This study hypothesizes that residents' sports literacy has a positive effect on residents' sports harvest and satisfaction.

The home-based fitness harvest draws the concept of “customer value perception.” Customer value perception refers to the overall evaluation of the product or service made by customers based on all their efforts and gains. The perception of home fitness gains refers to the evaluation of residents' physical and psychological feelings after home fitness practice. This feeling can be the physical relaxation of the exercise subject and psychological pleasure but can also be the emotional promotion between individuals participating in the exercise (10). The research believes that the gain of home fitness is an important factor in promoting residents' repeated participation in in-home fitness and that the gain of home fitness affects the satisfaction of home fitness to a large extent. The study hypothesizes that home-based fitness gains have a positive effect on home-based fitness satisfaction (11).

Residents' satisfaction with home fitness refers to residents' satisfaction with the “services” provided by society and their surroundings in the process of participating in in-home fitness. It is the psychological reaction of residents after they are satisfied with home fitness. Residents' satisfaction with home fitness is the target variable of this study. This study hypothesizes that residents' satisfaction with home fitness is affected by their intention to do home fitness, perception of social support, perception of the home fitness environment, residents' physical literacy, and gain from home fitness.

## Research objectives and methodology

In this study, 1,052 residents in 30 provinces and cities were surveyed by a self-designed questionnaire, and the data were collected to verify the conceptual model. As the subjects involved in the questionnaire were somewhat affected by the outbreak, the questionnaire channels of this study could only communicate through online channels such as the Internet and telephone (subjects completed questionnaires at home).

### Research objectives

In this study, a total of 1,194 questionnaires were sent out, and 1,052 were effective, with a rate of 88.1%, including 557 males, accounting for 52.7%, and 495 females, accounting for 47.1%. A total of 275 people aged 18–25 years accounted for 26.1%; 266 people aged 26–39 years accounted for 25.3%; 234 people aged 40–49 years accounted for 22.2%; 181 people aged 50–59 years accounted for 17.2%; 96 people aged 60 years and above accounted for 9.1%. A total of 412 people in the eastern part accounted for 39.2%, 386 people in the central part accounted for 36.7%, and 254 people in the western part accounted for 24.1%. There are 259 people with a high school education or less, accounting for 24.6%; 617 people with a college education, accounting for 58.7%; 176 people with a master's degree or above, accounting for 16.7%. In total, 95, or 9%, were cadres of state organs; 284, or 27%, were employees of enterprises and public institutions; 92, or 8.7%, were professional technicians; 126, or 12%, were production workers in agriculture, forestry, and animal husbandry; 74, or 7%, were self-employed; 297, or 28.2%, were students; and 84, or 8%, were others. Table 1 is the list of demographic characteristics of subjects (*n* = 1,052).

**Table 1:** List of demographic characteristics of subjects (*n* = 1,052).

**Variable**	**Frequency**	**Sample proportion (%)**	**Variable**	**Frequency**	**Sample proportion (%)**
**Gender**			**Professional**		
Male	557	52.9	Cadres of state organs	95	9.0
Female	495	47.1	Employees of enterprises and public institutions	284	27.0
Age (years)			Professional and technical personnel	92	8.7
18–25	275	26.1	Production personnel in agriculture, forestry, and animal husbandry	126	12.0
25–39	266	25.3	Self-employed	74	7.0
40–49	234	22.2	Students	297	28.2
50–59	181	17.2	Other	84	8.0
60 and above	96	9.1	Regional		
Record of formal schooling					
Senior high school and below	259	24.6	In the east	412	39.2
The university of	617	58.7	In the middle	386	36.7
Postgraduate or above	176	16.7	In the west	254	24.1

This study refers to the statistical table of the 2015 National Population Sample Survey published by the National Bureau of Statistics.

### Research methodology

The compilation of the questionnaire in this study is based on two aspects: first, by referring to the Chinese citizens' sports awareness survey report (2018) and related literature on the home fitness satisfaction survey, and combining it with the reality of home fitness during the epidemic, the interview outline was formulated [summing up the dimensions of the questionnaire of residents' satisfaction with home fitness during the period of the epidemic (participation degree of home fitness, the content of home fitness, space of home fitness venue, time, and frequency of participation in-home fitness)] and the second is to draw on the American Customer Satisfaction Model (ASCI) and the Chinese Customer Satisfaction Model (CSCI), formulate the contents of the questionnaire through interviews and revisions, and finally, determine the causal variables of the model based on home fitness intention, social sports culture support perception, home fitness environment perception, home fitness gain, and residents' sports literacy.

Based on the above content, writing the questionnaire on residents' fitness degree of satisfaction and its influencing factors at home during the outbreak of the COVID-19 epidemic, then eight experts reviewed the content of the questionnaire, the first draft of the questionnaire is identified after three rounds of modification, and try on a small scale questionnaire, with the respondents' feedback to the questionnaire of subject setting, the expression made revisions, the rationality and scientificity of the questionnaire are guaranteed. The revised questionnaire is divided into three parts. The first part is the demographic survey of the respondents, including gender, age, occupation, education background, and region. The second part is the questionnaire to assess residents' satisfaction with home fitness during the epidemic period. It uses 5-point Likert scale and sets 16 questions from four aspects, namely, sports participation, content, space, and time-frequency of home fitness. The third part is the questionnaire on influencing factors of residents' satisfaction with home fitness during the epidemic period. The 5-point Likert scale was also used to set 20 questions from five aspects, namely, home fitness intention, perception of social sports culture support, perception of home fitness environment, home fitness gain, and residents' physical literacy. SPSS22.0 and AMOS 24.0 were used for data processing.

## Research results and analysis

### Conceptual model analysis

Questionnaire reliability analysis mainly tests the consistency of each dimension of the questionnaire, which is generally measured by Cronbach's α coefficient. The consistency analysis of each dimension of this study is shown in Table 2. The Cronbach's α coefficient of each dimension is above 0.903, indicating that the questionnaire has high reliability.

**Table 2:** Consistency analysis of each dimension.

**The dimension**	**Cronbach's α coefficient**
Home fitness intention	0.916
Perception of social support	0.904
Home fitness environment perception	0.903
Residents' physical literacy	0.925
Home fitness gains	0.929
Satisfaction with home fitness	0.936

Correlation analysis was used to verify the structural validity of the questionnaire. The correlation coefficient between the factors of residents' satisfaction with home fitness and the total score ranged from 0.756 to 0.914, showing a high correlation, and the correlation coefficient between the factors ranged from 0.677 to 0.826, showing a moderate correlation, as shown in Table 3.

**Table 3:** Correlation coefficients of factors in the questionnaire of satisfaction with home fitness and between factors and the total score.

**Satisfaction with home fitness**	**Participation**	**Content**	**Floor space**	**Time and frequency**	**Total score**
Participation	1	-	-	-	-
Content	0.708**	1	-	-	-
Floor space	0.714**	0.782**	1	-	-
Time and frequency	0.677**	0.826**	0.742**	1	-
Total score	0.756**	0.914**	0.905**	0.896**	1

** Is a statistical symbol.

The correlation coefficient between the influencing factors of home fitness satisfaction and the total score ranged from 0.856 to 0.909, showing a high correlation, and the correlation coefficient between each factor ranged from 0.612 to 0.813, showing a moderate correlation, as shown in Table 4. It shows that all factors are correlated with each other and also have some independence, which can reflect the contents of the questionnaire to be investigated. Based on the above analysis results, it can be concluded that the questionnaire on residents' satisfaction with home fitness and its influencing factors during the epidemic period has good structural validity. Table 5 shows the confirmatory factor analysis of residents' satisfaction with the home fitness questionnaire during the epidemic period, showing that the fitting degree met the requirements.

**Table 4:** Correlation coefficients between factors and total scores of the questionnaire on influencing factors of residents' satisfaction with home fitness during the period of the epidemic.

	**Home Fitness Intention**	**Social support**	**The sports environment**	**Sports accomplishment**	**Sports to harvest**	**Total score**
Home fitness intention	1	-	-	-	-	-
Perception of social support	0.632**	1		-	-	-
Perception of sports environment	0.698**	0.612**	1	-	-	-
Residents' physical literacy	0.801**	0.624**	0.751**	1	-	-
Sports harvest for residents	0.813**	0.798**	0.794**	0.815**	1	-
Total score	0.886**	0.864**	0.881**	0.856**	0.909**	1

** Is a statistical symbol.

**Table 5:** The confirmatory factor analysis of residents' satisfaction with the home fitness questionnaire during the epidemic period showed that the fitting degree met the requirements.

**Indicators**	**Squared/df**	**GFI**	**RMSEA**	**RMR**	**CFI**	**NFI**	**NNFI**
The judgment standard	<3	>0.9	<0.10	<0.05	>0.9	>0.9	>0.9
Value	2.544	0.959	0.090	0.046	0.928	0.916	0.815

The study conducted correlation analysis on the questionnaire of influencing factors of residents' satisfaction with home fitness during the period of the epidemic, and the fitting degree met the requirements. Table 6 shows the fit degree of influencing factors of residents' satisfaction with home fitness during the epidemic period.

**Table 6:** Fit degree of influencing factors of residents' satisfaction with home fitness during the epidemic period.

**Indicators**	**Squared/df**	**GFI**	**RMSEA**	**RMR**	**CFI**	**NFI**	**NNFI**
The judgment standard	<3	>0.9	<0.10	<0.05	>0.9	>0.9	>0.9
Value	3.043	0.917	0.040	0.024	0.905	0.935	0.840

The questionnaire score of each subject, whether in the influence factors, home fitness intention and social support perception problem is higher, shows that the participation of residents at the home had a higher fitness intention at home, and on the outbreak during the social from all walks of life support for home fitness is more satisfied, and home fitness literacy, environmental awareness, and home fitness score are not very high; it shows that there are still a large number of residents who do not have the habit of home exercise and enough fitness knowledge. They are not very satisfied with the overall environment of home exercise, and the gain of home exercise is not very great. Table 7 shows the average value, standard deviation, and average score for each dimension.

**Table 7:** The average value, standard deviation, and the average score of each dimension.

**The dimension**	**The average**	**The standard deviation**	**The topic split**
Home fitness intention	14.88	2.41	3.72
Perception of social support	14.56	2.52	3.64
Home fitness environment perception	12.44	2.56	3.11
Residents' physical literacy	12.72	2.68	3.18
Home fitness gains	12.28	2.41	3.07
Participation	15.12	2.87	3.78
Content	12.56	2.65	3.14
Space equipment	12.16	2.51	3.04
Time and frequency	11.60	2.11	2.90

As can be seen from the satisfaction questionnaire, residents' sports participation scores are high, content and site equipment scores are average, and time and frequency scores are the lowest. It shows that the vast majority of residents participate in-home fitness but are not very satisfied with the content of exercise, exercise facilities, and equipment, and fail to form regular home fitness behavior.

### Hypothesis test

After a reliability and validity test and confirmatory factor analysis, the hypothesized model of this study was verified. After running the software, it is found that the fitting degree of the model does not meet the standard, and the path analysis results show that the hypotheses of H6 and H12 are not valid. Further modifications to the model are needed. Table 8 shows the test of significance of path coefficient of structural variables of a conceptual model.

**Table 8:** Test of significance of path coefficient of structural variables of a conceptual model.

**Assuming that**	**Path to the relationship between**			**P**	**Path coefficient**	**Conclusion 1** ***P* < 0.05**	**Conclusion 2** ***P* < 0.001)**
H1	Home fitness intention	->	Perception of social support	***	0.31	Support	Support
H2	Home fitness intention	->	Home fitness environment perception	***	0.32	Support	Support
H3	Home fitness intention	->	Residents harvest	***	0.37	Support	Support
H4	Residents' physical literacy	->	Home fitness intention	***	0.50	Support	Support
H5	Residents' physical literacy	->	Residents harvest	***	0.54	Support	Support
H6	Home fitness environment perception	->	Residents harvest	0.047	0.15	Support	Does not support
H7	Perception of social support	->	Residents harvest	***	0.36	Support	Support
H8	Perception of social support	->	Residents' satisfaction	***	0.44	Support	Support
H9	Home fitness environment perception	->	Residents' satisfaction	***	0.34	Support	Support
H10	Residents harvest	->	Residents' satisfaction	***	0.39	Support	Support
H11	Residents' physical literacy	->	Residents' satisfaction	***	0.47	Support	Support
H12	Home fitness intention	->	Residents' satisfaction	0.214	0.05	Does not support	Does not support

Note: -> indicates the path influence relationship, ***indicates significant at the level of P < 0.001, **indicates significant at the level of P < 0.05.

### Model modification

It is assumed that the reason why H6 is not completely true may be that some residents live in a poor home fitness environment, so residents do not feel the obvious impact of the home fitness environment on the harvest of home fitness. But given the outbreak period, residents' activity limitations, difficulty in obtaining normal home health supplies and equipment being unable to create a good fitness environment at home, and parts of the home fitness behavior, such as rope skipping, setting-up exercise, and treadmill running, is potentially kind of a nuisance, with the risk of affecting the relationship of neighbors. These factors in the particular historical period are likely to lead to a poor fitness environment at home for residents. However, with the end of the epidemic, people's demand for home fitness will be released, and the purchase of sports equipment will be convenient. Thus, residents are more likely to create a relatively satisfactory home fitness environment. Although the verification results indicate that residents' home fitness environment has no significant impact on the gain of home fitness (*P* = 0.047), it still has a certain impact. So, hypothesis H6 was not deleted in the correction.

The initial verification results show that residents' intention of home fitness has a certain negative impact on their satisfaction with home fitness, that is, the stronger the intention is, the lower the satisfaction will be. However, according to the analysis of *P* = 0.214, there is no significant relationship between residents' intention of home fitness and their satisfaction with home fitness. For this reason, delete H12.

**Figure 2:**
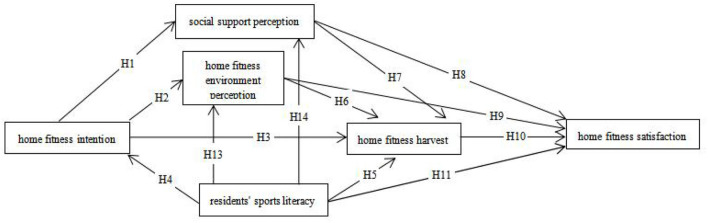
Modified model of residents' satisfaction with home fitness.

In the model modification index, it is found that there is a positive influence on residents' physical literacy and residents' perception of their home fitness environment and social support. To a certain extent, residents' physical literacy reflects the comprehensive level of sports skills, sports habits, and sports knowledge. Residents with higher sports literacy prefer to obtain their required home fitness skills from various channels, which would create a good home fitness environment, and people with higher sports literacy usually have purchased a certain amount of home fitness equipment. Therefore, this study believes that residents' physical literacy has a positive impact on residents' perception of the home fitness environment and social support. So, hypotheses H13 and H14 are added. Figure 2 shows the modified model of residents' satisfaction with home fitness. In summary, one hypothesis H12 is removed and two hypotheses H13 and H14 are added.

**Figure 3:**
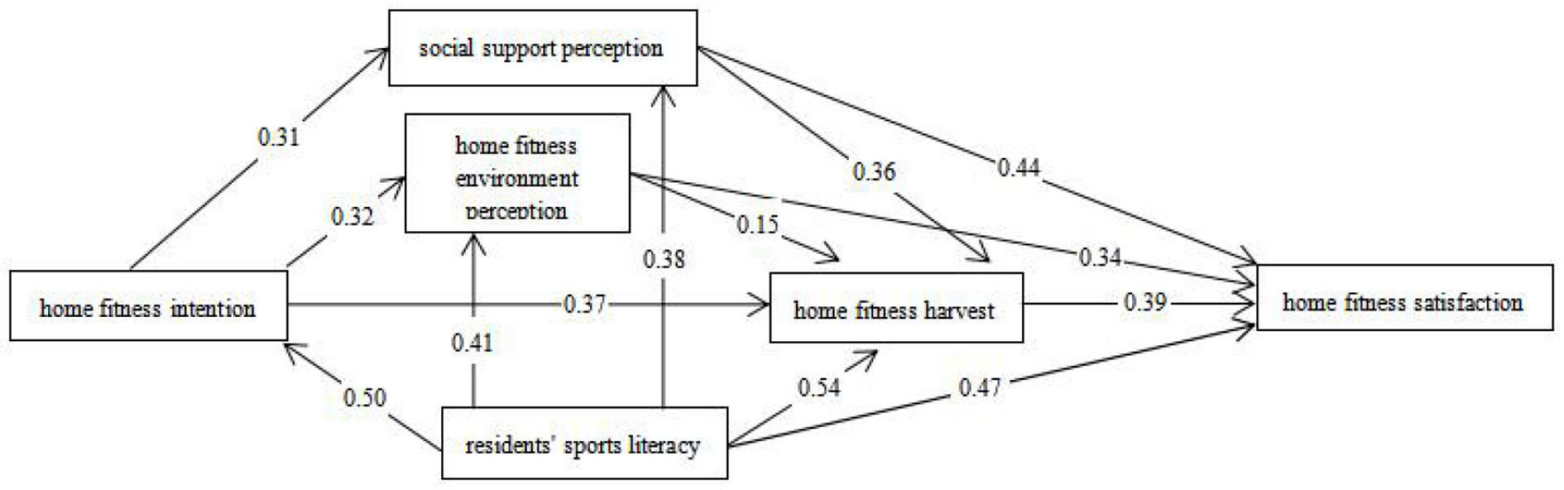
Satisfaction model of residents' home fitness.

After revision and re-examination, the model fitting degree index meets the standard, which indicates that the model is valid and has the significance of practical analysis. Finally, the satisfaction model of residents' home fitness is shown in Figure 3 (Table 9).

**Table 9:** Modified model variable fitting index.

**indicators**	**Squared/df**	**GFI**	**RMSEA**	**RMR**	**CFI**	**NFI**	**NNFI**
The judgment standard	<3	>0.9	<0.10	<0.05	>0.9	>0.9	>0.9
Value	2.11	0.906	0.037	0.024	0.923	0.915	0.941

Table 10 shows the path relationships of each factor. The results show that hypotheses H1, H2, H3, H4, H5, H6, H7, H8, H9, H10, H11, H13, and H14 are valid, but H6 is not supported. Since H6 has been explained, H12 is finally deleted and H13 and H14 are added.

**Table 10:** Test of significance of path coefficient of model structure variables.

**Assuming that**	**Path to the relationship between**			**P**	**Path coefficient**	**Conclusion 1 *P* < 0.05**	**Conclusion 2 *P* < 0.001)**
H1	Home fitness intention	->	Perception of social support	***	0.31	Support	Support
H2	Home fitness intention	->	Home fitness environment perception	***	0.32	Support	Support
H3	Home fitness intention	->	Residents harvest	***	0.37	Support	Support
H4	Residents' physical literacy	->	Home fitness intention	***	0.50	Support	Support
H5	Residents' physical literacy	->	Residents harvest	***	0.54	Support	Support
H6	Home fitness environment perception	->	Residents harvest	0.132	0.15	Support	Does not support
H7	Perception of social support	->	Residents harvest	***	0.36	Support	Support
H8	Perception of social support	->	Residents' satisfaction	***	0.44	Support	Support
H9	Home fitness environment perception	->	Residents' satisfaction	***	0.34	Support	Support
H10	Residents harvest	->	Residents' satisfaction	***	0.39	Support	Support
H11	Residents' physical literacy	->	Residents' satisfaction	***	0.47	Support	Support
H13	Residents' physical literacy	->	Home fitness environment perception	***	0.41	Support	Support
H14	Residents' physical literacy	->	Perception of social support	***	0.38	Support	Support

Note: -> indicates the path influence relationship, ***indicates significant at the level of P < 0.001, **indicates significant at the level of P < 0.05.

## Discussion

This study validated the satisfaction model of home fitness with structural equations and revealed the relationships among the intention of home fitness, perceived social support, perceived environment of home fitness, a gain of home fitness, residents' physical literacy, and satisfaction with home fitness. Findings (1) During the period of the epidemic at home, residents' enthusiasm for home fitness remained unchanged, online services provided strong social support, and the home fitness environment had a large room for improvement. Some residents failed to develop regular home fitness habits. During the outbreak, the sedentary lifestyle at home and the external pressure from the outbreak made the residents realize the importance of fitness and physical and mental health. Plenty of people are actively involved in home fitness, and at all levels of government, schools, media, sports departments, and service agencies have launched home fitness programs. In addition, pictures and text, video, and broadcast ways to promote fitness at home have been achieved successfully, but residents' home fitness environment still needs to be improved (12). Residents' participation degree in home fitness is high, but the time and frequency of participation are not satisfactory, which indicates that a large number of residents do not develop the habit of home fitness. (2) The perception of social support and home fitness environment have a positive impact on residents' home fitness harvest and satisfaction with home fitness. During the epidemic period, home fitness gained unprecedented social support. Professors and doctoral supervisors went online to popularize fitness knowledge, and physical education teachers became “18-line anchors,” while handsome men and women from various sports service agencies opened online live-streaming fitness courses. People are creative and use everyday objects to create a fitness environment at home. For example, rice bags can be used instead of kettlebells, balloons can be used as air volleys, and tables can be used as table tennis tables (13). The more social support residents get, the better the home fitness environment will be; the more residents gain from home fitness, the higher their satisfaction with home fitness will be. (3) Residents' physical literacy directly affects residents' intention to exercise at home, perception of the environment of exercise at home, perception of social support, the harvest of exercise at home, and satisfaction with exercise at home (14). To a great extent, the level of physical literacy determines the size of a person's scientific fitness ability. The study found that physical literacy had a positive impact on the intention to exercise at home, the perception of social support, the perception of the environment of exercise at home, the harvest of exercise at home, and the satisfaction with exercise at home. The higher the sports quality, the more they can understand the benefits of physical fitness; the more people gain the benefits of physical fitness, the higher the loyalty to physical exercise will be; and good sports attainments can help to have a better channel to gain fitness knowledge, choose to science for their own home, and know how to make and use their living environment to create a satisfied and comfortable fitness environment at home with satisfaction.

According to the results of this study, during the outbreak of residents at home, the satisfaction degree of fitness is not high, especially the social support score with home fitness environment is not very outstanding. The major cause is that, on the one hand, physical sports consumption has been affected by the epidemic, which to a certain extent causes some residents to be unsatisfied with the exercise environment at home. However, after the end of the epidemic, physical sports consumption is expected to peak, especially home fitness equipment consumption. Persistence is the most important thing in sports and fitness. Although more people participated in the in-home exercise, the time and frequency of the exercise do not meet the expectations. First, the communication and guidance methods of home exercise content have been affected to some extent in the epidemic, and home exercise can only be promoted through offline channels such as the Internet and TV. Second, the content and form of home fitness are limited to a certain extent, which makes it difficult for some providers of home fitness content to find suitable shooting sites and filming equipment, and effectively organize filming personnel and logistics support personnel. Moreover, with the fierce outbreak of the epidemic, the time for shooting, organizing, and planning is relatively limited. Finally, such a large fitness activity at home is the first time to appear, so it takes time to get into the habit of home fitness. And scenes, space, during the outbreak of the above factors are affected by a certain, so residents fitness time and frequency are not high also understandable. It is believed that after the outbreak, with the recovery of the social order and continuous improvement of the high-quality fitness services, content at home and satisfaction with fitness at home will increase.

The emergence of the epidemic has forced departments at all levels, schools, sports enterprises, sports institutions, and others to think about solutions to home fitness. The importance of home fitness has received unprecedented attention from the whole society. Affected by various isolations and restrictions during the epidemic, the emerging intelligent fitness scenes and equipment have attracted much attention. In the future, home-based fitness will be characterized by diversification, intelligence, individualization, and entertainment. Internet, big data, 5G, and cloud technology will play an important role in the change. The outbreak of the COVID-19 epidemic is not only a heavy blow for the sports fitness service industry and equipment manufacturing industry but also an opportunity for reform. The outbreak will deeply affect people's lifestyles. Sports service departments should speed up the use of innovative technologies to build a new national fitness service platform and promote the upgrading and improvement of national fitness services.

Sports service departments should innovate their approaches to sports service, adopt the combination of online and offline approaches, and vigorously promote the establishment of digital and intelligent home fitness service mechanisms. Change the way of work; gradually change from offline to offline and online combination; gradually expand the path of sports and fitness services based on grassroots, community, family, and cooperation; deepen technological innovation; further, increase the guarantee of home fitness service funds; and build and constantly improve the home fitness service mechanism.

## Conclusion

The sudden epidemic has caused great trauma to the mental health of people, especially those in the worst-hit areas and medical workers. In this period, sports have played a unique and irreplaceable role in regulating the psychological health of people. Electronic publications such as home fitness manuals have a calming and soothing effect. The square cabin hospital, transformed from the stadium, also played a significant role in the fight against the epidemic. After the end of the epidemic, residents' sports consumption will usher in a new upsurge, and the upstream and downstream of the sports industry chain will absorb a large number of workers, which has played a positive role in social stability. At present, it is a critical period for China to realize sports power and build a healthier China. The outbreak makes people and the government realize the importance of physical fitness, which provides opportunities to discover and meet the needs of residents' sports fitness. The outbreak is also a good time to improve the public's sports attainments. Home exercise cannot rely only on the spontaneous behavior of the individual. Each department should better seize the opportunity to vigorously promote the national fitness and healthy life concept. At the same time, scientific knowledge of fitness should be publicized and actively encourage residents to form the habit of lifelong exercise, thus creating a fitness atmosphere as well as comprehensively improving the sports literacy of people.

## Data availability statement

The original contributions presented in the study are included in the article/supplementary material, further inquiries can be directed to the corresponding author.

## Author contributions

DC and BZ jointly conducted a statistical analysis on the model of this article and conducted statistics together. BZ completed the frame writing of the entire article. DC supplemented the data, arranged the data, and polished the article. Both authors contributed to the article and approved the submitted version.

## Funding

This research study was sponsored by the General Project of 2020 Zhejiang Philosophy and Social Science Planning: Thrift Management Model of Large-Scale Sports Events and enlightenment to Hangzhou Asian Games (Project No. 20NDJC226YB); The Fundamental Research Funds of Shandong University (Project No. 2020GN029); Shandong Social Science Planning and Research Project (Project No. 21DTYJ02); 2022 Shaoxing Higher Education Teaching Reform Research Project; and 2021 Zhejiang College of Construction Technology Innovation Team: Asian Olympic Culture Research Team (No. 2021.45.9B).

## Conflict of interest

The authors declare that the research was conducted in the absence of any commercial or financial relationships that could be construed as a potential conflict of interest.

## Publisher's note

All claims expressed in this article are solely those of the authors and do not necessarily represent those of their affiliated organizations, or those of the publisher, the editors and the reviewers. Any product that may be evaluated in this article, or claim that may be made by its manufacturer, is not guaranteed or endorsed by the publisher.

## References

[1] Woon FAC Omkar D Aaker JL Aboltins K Rivza B Adusei C. Effectiveness of loyalty programs in customer retention: a multiple mediation analysis. Jindal J Bus Res. (2021) 10:7–32. 10.1177/22786821211000182

[2] Fei D Xiaoxi W Xiaonan L. A model for the satisfaction of pre-school students' education practice based on structural equation. Preschool Educ Res. (2018) 2018:36–45. 10.13861/j.cnki.sece.2018.10.004

[3] Haiyan H Yikun K. Influence of the fit between sports events and city image on audience satisfaction and revisit intention. China Sports Sci Technol. (2018) 54:12–20. 10.16470/j.csst.201804002

[4] Zhili C Mina L Xuebing L Lei Z Wei Y Chengqian X. Research on public sports resources, sports participation and public sports service satisfaction. Sports Sci. (2016) 37:77–87.29877672

[5] Saez I Solabarrieta J Rubio I. Motivation for physical activity in university students and its applications relation with gender, amount of activities, and sport satisfaction. Sustainability. (2021) 6:3183. 10.3390/su13063183

[6] Li X Che L Yuan J Guo S Wang K. Physical exercise activities, medical consumption and health satisfaction: a survey based on the sports participation of Beijing-Tianjin-Hebei Urban Residents. J Wuhan Inst Phys Educ. (2019) 53:34–42. 10.15930/j.cnki.wtxb.2019.07.005

[7] Zhang Y. Study on the sociological constraints of urban residents' exercise and fitness behavior. J Beijing Sports Univ. (2019) 42:85–94. 10.19582/j.cnki.11-3785/g8.2019.07.010

[8] Wang X. Frontier hotspots, topic clustering and expansion space of international sports literacy research. J Beijing Sport Univ. (2019) 42:102–16. 10.19582/j.cnki.11-3785/g8.2019.10.012

[9] Sheng PN Xiang CP Yun CP Wen WZ. Home fitness and rehabilitation support system implemented by combining deep images and machine learning using unity game engine. Sensor Mater. (2022) 34:1971–90. Available online at: https://sensors.myu-group.co.jp/sm_pdf/SM2947.pdf

[10] Arne JP. Hidden in plain sight: the spatial and industrial logics of home fitness technologies. New Rev Film Television Stud. (2021) 19:485–509. 10.1080/17400309.2021.1960099

[11] Hung CJ Chang HM Lin CJ Yen TF. Modelling the indicators of environmental risk for fitness activities. Asian J Educ Soc Stud. (2020). 10.9734/ajess/2020/v6i13016726892339

[12] Nyenhuis SM Greiwe J Zeiger JS Nanda A Cooke A. Exercise and fitness in the age of social distancing during the COVID-19 pandemic. J Allergy Clin Immunol Pract. (2020) 8:2152–5. 10.1016/j.jaip.2020.04.039 32360185 PMC7187829

[13] Furjan Mandic G Bilbija B Radas J Ivkovic G. Impact of home fitness program on anthropological characteristics of physically active and physically inactive people. Sport Mont. (2018). 10.26773/smj.180207

[14] Zhu Q Sun J. Evaluation on home fitness and community sports activities based on network survey. Int J Smart Home. (2016) 16:33-6. 10.14257/ijsh.2016.10.5.29

